# Parental Reports of Oral Health Behaviours and Dental Caries in Romanian Children and Adolescents: A Cross-Sectional Study

**DOI:** 10.3390/children13091154

**Published:** 2026-08-27

**Authors:** Darius Dacian Macavei, Raluca Iurcov, Abel Emanuel Moca, Rebeca Daniela Marton, Gabriela Ciavoi, Ligia Luminița Vaida

**Affiliations:** 1 Doctoral School of Biomedical Sciences, University of Oradea, 1 Universitatii Street, 410087 Oradea, Romania; macavei.dariusdacian@student.uoradea.ro (D.D.M.); rebeca.marton@uoradea.ro (R.D.M.); 2 Department of Dentistry, Faculty of Medicine and Pharmacy, University of Oradea, 4 Universității Street, 410087 Oradea, Romania; riurcov@uoradea.ro (R.I.); gciavoi@uoradea.ro (G.C.); ligia_vaida@uoradea.ro (L.L.V.)

**Keywords:** dental caries, oral hygiene, children, oral health behaviour

## Abstract

**Background/Objectives**: Dental caries remains one of the most prevalent chronic diseases in childhood, influenced by a combination of hygiene, dietary, and access-related behaviours that are largely shaped by parental involvement. This study aimed to evaluate oral hygiene habits, dietary risk factors, access to dental services, awareness of caries prevention, and the impact of oral health on the child, in relation to parent-reported dental caries in Romanian children and adolescents. **Methods**: A cross-sectional study was conducted using a structured online questionnaire completed by parents or legal guardians of 508 children and adolescents aged under 18 years. Associations between categorical variables and reported caries were assessed using chi-square tests and Cramér’s V. Ordinal variables were compared using the Mann–Whitney U test. A multivariable logistic regression identified independent predictors of reported caries, with robustness examined through sensitivity analyses. **Results**: Parent-reported caries was present in 61.2% of children. The strongest associations with caries were the absence of parental supervision during brushing (Cramér’s V = 0.364), the age at which children began brushing unsupervised (V = 0.354), and the reason for the last dental visit (V = 0.426). In the multivariable model, older age, absence of brushing supervision, sweets before bedtime, less frequent dental visits, and rural residence were independent predictors (all *p* < 0.05; Nagelkerke R^2^ = 0.361). All six assessed dimensions of oral-health impact on the child were significantly associated with caries, with physical discomfort showing the largest effect (V = 0.349). **Conclusions**: Parent-reported caries risk in this population clustered around a small number of modifiable behaviours rather than any single dominant factor, while reported caries was associated with a substantial burden on children’s daily functioning. These findings point to sustained parental supervision and routine, non-symptomatic dental care as priority targets for future prevention efforts, although intervention studies are needed to confirm their effectiveness.

## 1. Introduction

Children’s oral health is influenced by a combination of biological, behavioral, familial, and social factors. During childhood, habits related to oral hygiene, diet, and dental visits are not yet fully autonomous but depend largely on the supervision and decisions of parents or caregivers. For this reason, evaluating the daily behaviors of the child and family can provide important information for understanding the risk of dental caries [1].

Oral diseases remain a major public health problem, and dental caries is still one of the most common oral conditions worldwide. The World Health Organization emphasizes that oral conditions affect a very large number of people and can have significant consequences for general health, quality of life, and access to healthcare services [2]. In children, dental caries is important not only because of its frequency but also because it can be prevented through simple, consistently applied measures: correct oral hygiene, control of sugar consumption, use of fluoride products, and regular dental check-ups [3,4].

Dental caries has a multifactorial etiology. Frequent consumption of sweets, unhealthy snacks, and sugar-sweetened beverages contributes to an increased cariogenic risk, especially when these habits are associated with insufficient or irregular toothbrushing. Recent evidence shows that intake of unhealthy foods and beverages is associated with a higher risk of dental caries in children [5]. Moreover, consuming sweet foods or drinks before bedtime can be particularly unfavorable, as salivary flow decreases during sleep, reducing the natural protective mechanisms of the oral cavity [6].

Oral hygiene habits are a central element of prevention. Brushing frequency, duration, brushing before bedtime, use of fluoride toothpaste, periodic replacement of the toothbrush, and use of auxiliary oral hygiene methods can all influence caries risk. In children, these behaviors are often dependent on parental involvement, particularly through supervision of brushing and the establishment of a daily routine. Studies on parental behaviors show that parents’ attitudes, beliefs, and level of knowledge can directly influence their children’s oral health [7,8].

Oral health literacy also plays an important role. Parents who better understand the causes of caries, the role of fluoride, the importance of regular check-ups, and the effects of cariogenic diet can more easily adopt preventive behaviors for their children. A recent literature analysis showed that parental oral health literacy is associated with children’s oral health outcomes [9]. However, the mere existence of information does not guarantee its application. Preventive behaviors can be influenced by available time, treatment costs, access to dental services, fear of the dentist, or parents’ perception of the severity of dental problems [10].

Access to dental services is another important aspect. Regular visits allow early identification of carious lesions, the application of preventive measures, and the education of the child and family. In contrast, seeking dental care only in cases of pain or complications reflects a late, predominantly curative approach. In such situations, the child may require more complex treatment, and the dental experience may be perceived negatively [11]. In Romania, studies on pediatric dental emergencies have shown that untreated conditions, including dental caries, can contribute to children presenting for emergency care [12].

The consequences of dental caries are not limited to damage to dental structures. Pain, sensitivity, difficulties chewing, avoidance of certain foods, sleep disturbances, embarrassment about the appearance of teeth, and difficulties in social interaction can all affect the child’s daily life. Studies on oral health–related quality of life have shown that the presence of caries can negatively affect both children and their families [13,14]. For this reason, evaluating the functional, physical, psychological, and social impact of dental problems offers a more complete perspective on the importance of prevention.

In the current context, data are needed that describe not only the presence of dental caries but also the associated behavioral and social factors. In Romania, recent research has shown that certain family characteristics, such as educational level and family structure, can influence parental behaviors regarding caries prevention in children [15]. Nevertheless, it remains important to analyze several dimensions in an integrated manner: oral hygiene, diet, access to dental services, the level of awareness regarding prevention, and the impact of oral health on the child. While the individual associations between parental supervision, dietary habits, dental attendance, and childhood caries are already well established internationally, such integrated, multi-domain evidence combining these dimensions within a single analytical framework remains scarce for the Romanian pediatric population, a gap this study seeks to address.

Addressing this gap, the aim of the present study was to evaluate oral hygiene habits, dietary risk factors, access to dental services, the level of awareness regarding caries prevention, and the impact of oral health on the child, in relation to the reported diagnosis of dental caries. The study sought to identify factors associated with parent-reported caries in order to support the development of educational and oral health prevention programs targeting children, parents, and the community.

## 2. Materials and Methods

### 2.1. Study Design and Participants

An observational, cross-sectional study was conducted using a structured questionnaire addressed to the parents or legal guardians of children. The study aimed to assess oral hygiene habits, dietary risk factors, access to dental services, the level of awareness regarding caries prevention, and the impact of oral health on the child.

Data were collected between 1 April and 1 July 2026 through an online questionnaire created in Google Forms (Google LLC, Mountain View, CA, USA). The questionnaire was administered in Romanian and the link was distributed electronically via social media and parent-oriented groups to facilitate access to the target population in Romania. Participation was voluntary and anonymous. Before accessing the questionnaire, participants were shown an introductory page describing the study and their right to withdraw at any time, and were required to actively confirm their consent before proceeding to the questionnaire. For a questionnaire to be considered valid for analysis, responses were required to be complete across all items. Questionnaires with one or more unanswered items were identified during data screening and excluded. The platform’s built-in restriction (one response per Google account) was used to prevent duplicate submissions.

**Figure 1:**
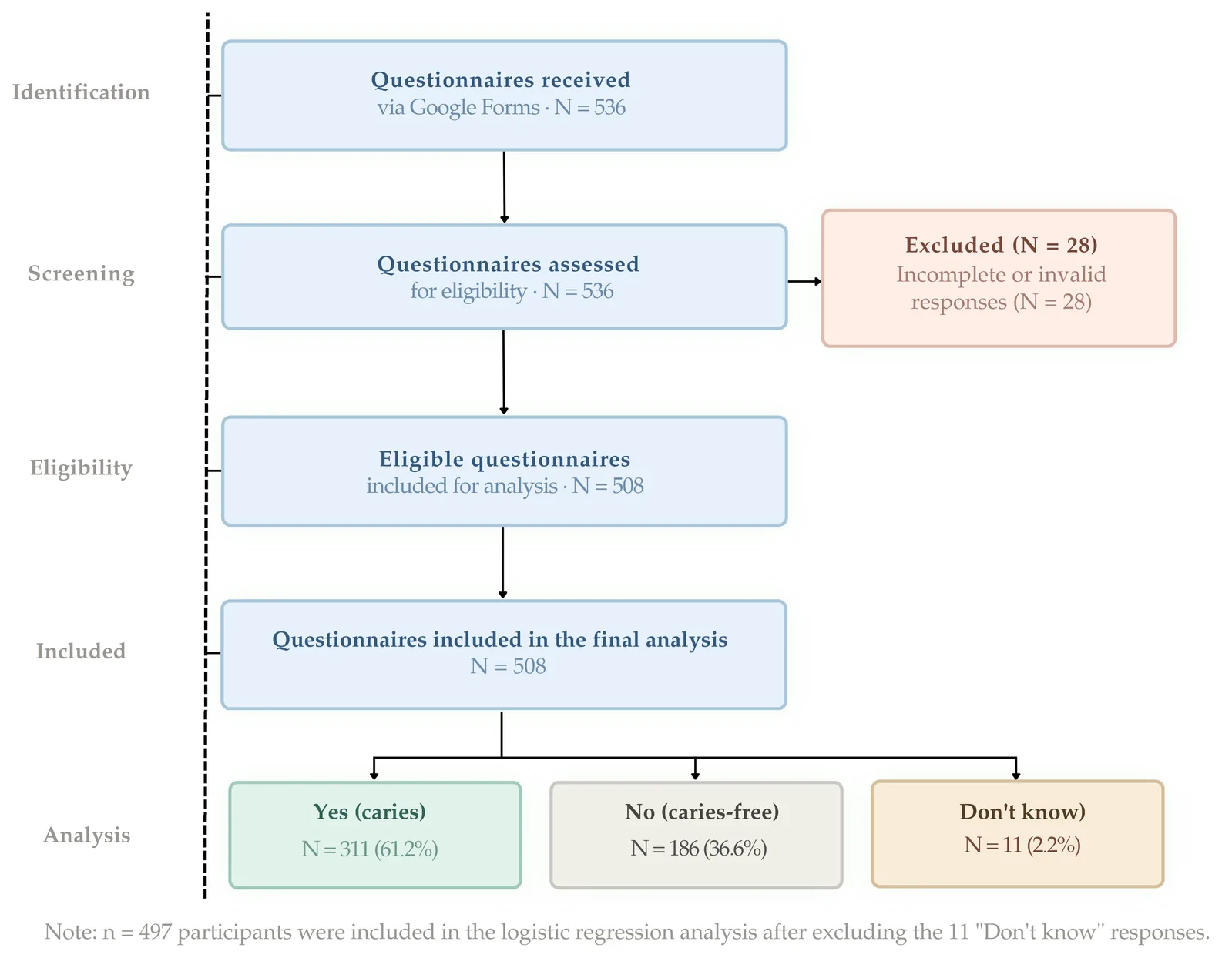
Flow diagram of the questionnaire selection process (2026).

Inclusion criteria required the respondent to be the parent or legal guardian of a child aged under 18 years residing in Romania; for families with more than one eligible child, parents were asked to report on their youngest child. Each questionnaire was completed by a single parent or guardian and referred to a single child. A total of 536 responses were received. The sole exclusion criterion was incompleteness, defined as one or more unanswered items; 28 questionnaires were excluded on this basis. The selection process is presented in Figure 1. After applying these inclusion and exclusion criteria, the final sample comprised 508 valid questionnaires.

### 2.2. Questionnaire and Study Variables

The research instrument was a structured questionnaire, developed in Romanian, designed to assess factors associated with children’s oral health. The questionnaire comprised items with predefined responses, organised into six distinct sections:

Section I—General information about the child: age, sex, place of residence, county, parent-reported diagnosis of dental caries, history of dental extractions, and use of an orthodontic appliance;

Section II—Oral hygiene habits: frequency and duration of brushing, brushing before bedtime, supervision of brushing, type of toothbrush and toothpaste, and auxiliary oral-hygiene methods;

Section III—Diet and risk factors: frequency of consumption of sweets, unhealthy snacks and sweetened beverages, consumption of sweets before bedtime, and type of drink consumed between meals;

Section IV—Access to dental services: frequency of check-ups, reason for the last visit, dental fear, perceived access to dental services, and receipt of free treatments;

Section V—Awareness and caries prevention: discussions with the child about oral health, sources of information, educational programmes, and community prevention campaigns;

Section VI—Perceived impact of oral health on the child: parent-reported functional limitations, physical discomfort (dental pain over the preceding three months), psychological, social, sleep and school/daily-activity impacts.

Prior to data collection, the questionnaire was piloted on a convenience sample of 30 parents to assess item clarity, comprehensibility, and completion time, and was reviewed for content and face validity by specialists in pediatric dentistry. Item wording was refined based on this feedback.

### 2.3. Sample Size

The minimum required sample size was calculated for a cross-sectional study, assuming a 95% confidence level and a 5% margin of error. Using a conservative proportion of *p* = 0.5, the estimated minimum size was approximately 384 participants. The final sample included 508 valid questionnaires, exceeding this requirement.

### 2.4. Statistical Analysis

Data were organised and processed in Microsoft Excel (Microsoft Corporation, Redmond, WA, USA), and statistical analysis was performed using IBM SPSS Statistics version 25.0 (IBM Corp., Armonk, NY, USA). Categorical variables were expressed as absolute frequencies and percentages. Associations between categorical variables were assessed using the chi-square test or Fisher’s exact test, and the strength of associations using Cramér’s V coefficient. For multiple-choice items, each response option was tested individually against reported caries status (selected vs. not selected). Ordinal dietary-frequency variables (Section III) were additionally compared using the Mann–Whitney U test, given their fine-grained response scales. For these variables, Cramér’s V was computed separately from the Pearson chi-square statistic of the corresponding contingency table (ordinal category × reported caries status), to provide a standardized effect-size measure comparable across all bivariate analyses in the study. The Mann–Whitney U test provided the reported *p*-value, and Cramér’s V was not used for significance testing. Given the exploratory nature of these bivariate analyses and the large number of comparisons performed, no correction for multiple testing (e.g., Bonferroni) was applied. *p*-values were interpreted alongside Cramér’s V effect sizes, and confirmatory inference was reserved for the multivariable model.

To identify independent predictors of the parent-reported caries diagnosis, a binary logistic regression analysis was performed (dependent variable: caries Yes = 1, No = 0; *n* = 497, excluding the 11 respondents who answered “Do not know”). The independent variables entered were: child age, sex, place of residence, brushing supervision, frequency of consumption of sweets before bedtime, frequency of dental visits, and type of toothpaste. Age was grouped into four categories corresponding to recognised stages of dental development: primary dentition (under 6 years), early mixed dentition (6–9 years), late mixed dentition (10–12 years), and permanent dentition (13–18 years), rather than into equal-width intervals, reflecting clinically meaningful transitions rather than a purely statistical partition. Brushing supervision was recoded as a binary variable (absence vs. presence of parental/family supervision); child age, frequency of sweets before bedtime, and frequency of dental visits were entered as ordinal variables; residence, toothpaste type, and sex were entered as binary variables (rural vs. urban; non-fluoride vs. fluoride, with “does not use” and “do not know” responses classified as non-fluoride; male vs. female). The reason for the last dental visit, despite showing the strongest bivariate association with caries (Cramér’s V = 0.426), was not entered into the multivariable model, as two of its response categories (‘dental pain’ and ‘caries treatment’) directly reference a caries-related complaint and would therefore risk reverse causation. Independent variables were selected a priori based on plausibility as antecedent, modifiable exposures rather than on bivariate significance alone, which is why sex and toothpaste type were retained despite not reaching significance. Results are expressed as odds ratios (OR) with 95% confidence intervals. Model adequacy was assessed using the chi-square test and the Nagelkerke R^2^ coefficient. The robustness of the model was further examined in a sensitivity analysis using alternative specifications for the handling of “Do not know” responses and model parsimony. The threshold for statistical significance was set at *p* < 0.05.

In addition, the internal consistency of each section containing ordinal-response items was assessed by calculating Cronbach’s alpha (α). Cronbach’s alpha was calculated as a descriptive check of item coherence within each section, rather than as a precursor to constructing composite scores. All analyses treated items individually throughout. The α values obtained support this approach, as internal consistency did not reach levels that would justify pooling items into a single score, consistent with the descriptive-epidemiological aim of capturing distinct behaviours rather than a unidimensional construct. Because the questionnaire is descriptive-epidemiological in nature, quantifying distinct behaviours and attitudes rather than a single latent construct, the α values obtained were interpreted with two well-established methodological considerations in mind: Cronbach’s alpha is sensitive to the number of items, with short scales (here, 4–6 items per section) tending to yield lower coefficients even with adequate inter-item correlation; and alpha assumes unidimensionality, so sections deliberately covering distinct, non-redundant behaviours rather than a single latent construct are expected to show comparatively lower internal consistency [16]. Accordingly, low-to-moderate α values were not treated as evidence against the adequacy of the instrument, but as consistent with its descriptive, multidimensional design. The α coefficients obtained were: Section II (oral hygiene, 6 ordinal-response items) α = 0.531 (*N* = 473); Section III (diet, 4 items) α = 0.470 (*N* = 508); and Section IV (access to dental services, 4 items) α = 0.619 (*N* = 508). Section VI items were analysed individually rather than as a composite scale, and internal consistency was therefore not calculated. Sections I and V contained no items suited to this calculation and were likewise excluded.

### 2.5. Ethical Considerations

The study was conducted in accordance with the ethical principles of the Declaration of Helsinki (1964) and its subsequent amendments. The protocol was approved by the Ethics Committee of the University of Oradea (IRB No. 4594/31.03.2026). Participation was voluntary and anonymous, and informed consent was obtained from all respondents prior to data collection.

## 3. Results

### 3.1. Characteristics of the Study Sample

A total of 508 valid questionnaires completed by parents or legal guardians were analysed. Boys accounted for 54.3% (*n* = 276) and girls for 45.7% (*n* = 232). Children under 6 years formed the largest age group (36.4%, *n* = 185), followed by the 6–9-year group (27.4%, *n* = 139), the 13–18-year group (21.9%, *n* = 111) and the 10–12-year group (14.4%, *n* = 73). Most children resided in urban areas (78.5%, *n* = 399). A parent-reported diagnosis of dental caries was present in 61.2% of children (*n* = 311), 14.2% (*n* = 72) had undergone at least one dental extraction, and 10.8% (*n* = 55) wore an orthodontic appliance (Table 1).

### 3.2. Oral Hygiene Behaviours

Just over half of the children brushed twice a day or more (52.4%, *n* = 266), and fluoride toothpaste was used by 62.0% (*n* = 315). Most children (74.1%, *n* = 368) started brushing their teeth unsupervised before the age of 6, and 56.7% (*n* = 282) had their toothbrush replaced every three months or more often. Parental supervision of brushing, brushing before bedtime, toothpaste type, the age at which the child began brushing alone, and the parent’s perceived adequacy of the child’s hygiene habits were each significantly associated with parent-reported caries. The strongest hygiene-related association was the absence of supervision (Cramér’s V = 0.364, *p* < 0.001), closely followed by the age at which the child started brushing alone (V = 0.354, *p* < 0.001), then brushing before bedtime (V = 0.190, *p* = 0.003), toothpaste type (V = 0.182, *p* < 0.001), and perceived adequacy of hygiene habits (V = 0.164, *p* = 0.004). Among auxiliary hygiene methods, use of mouthwash (V = 0.167, *p* < 0.001) and not using any auxiliary method (V = 0.181, *p* < 0.001) were both significantly associated with parent-reported caries, while use of dental floss was not (*p* = 0.312). Toothbrushing frequency, brushing duration, toothbrush replacement frequency, and rinsing with water/mouthwash after meals were not significantly associated with reported caries (Table 2).

### 3.3. Dietary Habits

Consumption of sweets and unhealthy snacks was frequent: 18.5% of children (*n* = 94) consumed them several times a day and 31.3% (*n* = 159) once a day. Water was the predominant between-meal drink (88.2%, *n* = 448). Most parents (61.4%, *n* = 305) believed that diet influences their child’s oral health “very much,” though this perception itself was not significantly associated with reported caries (*p* = 0.138). All other dietary variables showed a significant dose-dependent association with reported caries. Consumption of sweets before bedtime showed the strongest gradient (Cramér’s V = 0.312, *p* < 0.001), followed by sweetened beverages (V = 0.278), sweets and snacks (V = 0.231), fresh fruit and vegetables (V = 0.212), and type of between-meal drink (V = 0.175, *p* = 0.009) (Table 3).

### 3.4. Access to and Use of Dental Services

The reason for the last dental visit showed the strongest association with parent-reported caries of any variable in the study (Cramér’s V = 0.426, *p* < 0.001), followed by referral to a specialist (V = 0.273), the timing of the last visit (V = 0.307), and check-up frequency (V = 0.293). Access to free public treatments was also significant (V = 0.264), as were the reasons reported for not attending the dentist regularly: not perceiving any dental problems was the strongest of these (V = 0.264, *p* < 0.001), followed by the child’s fear of the dentist (V = 0.168, *p* < 0.001) and high treatment costs (V = 0.148, *p* = 0.001); lack of available time, difficult access to a dental office, and other reasons were not significant. Dental fear itself showed a weak association (V = 0.128, *p* = 0.017). Perceived ease of access to services was not significantly associated with caries (Table 4).

### 3.5. Awareness and Caries-Prevention Behaviours

Most parents reported having discussed the importance of oral health and caries prevention with their child, either frequently (76.3%, *n* = 379) or occasionally (19.7%, *n* = 98), though this was not significantly associated with reported caries (*p* = 0.332). The dentist was the most influential source of oral health information significantly associated with reported caries (V = 0.211, *p* < 0.001), followed by parents/family (V = 0.113, *p* = 0.012); school/kindergarten and mass media were not significant sources. Just over half of the children (54.1%, *n* = 269) had participated in an educational program on oral hygiene, though this association did not reach significance (*p* = 0.096). Parents’ views on the most effective educational method showed no significant association with reported caries for any option. Most parents felt local prevention campaigns were insufficient (65.6%, *n* = 326) and expressed interest in participating in a school- or community-based prevention program (73.4%, *n* = 365), but neither perception was significantly associated with reported dental caries (Table 5).

### 3.6. Perceived Oral-Health Impact on the Child

All six impact dimensions were significantly associated with reported caries, forming the strongest and most consistent block of associations in the entire dataset. Physical discomfort showed the largest effect: 26.4% of children (*n* = 131) had reported dental pain or sensitivity in the preceding three months, with 13.5% (*n* = 67) avoiding certain foods because of it (V = 0.349, *p* < 0.001). Functional limitations were reported in 19.1% of children (*n* = 95, V = 0.304, *p* < 0.001). Psychological effects were also common: 17.8% of children (*n* = 88) had felt embarrassed about their teeth, with 8.5% (*n* = 42) additionally reporting reduced self-confidence (V = 0.263, *p* < 0.001). Social handicap affected 11.0% of children (*n* = 55, V = 0.208, *p* < 0.001). Dental problems affected sleep in 17.6% of children (*n* = 87, V = 0.194, *p* < 0.001) and school performance in 5.4% (*n* = 27, V = 0.177, *p* = 0.001) (Table 6).

### 3.7. Multivariable Logistic Regression and Sensitivity Analysis

A multivariable binary logistic regression identified independent predictors of reported caries. The model was statistically significant (χ^2^(7) = 152.69, *p* < 0.001; Nagelkerke R^2^ = 0.361). Five variables were independent predictors: older child age (OR = 1.84; 95% CI 1.44–2.36), absence of brushing supervision (OR = 2.11; 95% CI 1.22–3.63), consumption of sweets before bedtime (OR = 1.76; 95% CI 1.44–2.15), frequency of dental visits (OR = 1.47; 95% CI 1.23–1.76) and rural residence (OR = 1.86; 95% CI 1.06–3.25); child sex and non-fluoride toothpaste were not independent predictors. The robustness of these findings was tested across four alternative specifications differing in the handling of the 11 “Do not know” caries responses and in model parsimony. The four core predictors remained significant with stable effect sizes in every specification, whereas rural residence lost significance when “Do not know” responses were reclassified as absence of caries (OR = 1.63; 95% CI 0.95–2.79), indicating that this association is the most sensitive to outcome definition (Table 7).

## 4. Discussion

In this cross-sectional sample of 508 children and adolescents, the factors most consistently associated with parent-reported caries were not isolated behaviours but ones tied to how much adult oversight and preventive care surrounded the child. The absence of supervision during brushing showed the strongest bivariate association in the entire dataset after the impact variables, and remained an independent predictor in the multivariable model. The age at which children began brushing unsupervised followed a similar pattern, suggesting that an early, unaided transition to independent brushing may carry comparable risk to lack of supervision itself. This would be broadly consistent with evidence from younger age groups, where Castilho et al. found that even minimal parental involvement in brushing, occurring as infrequently as once a day on some days of the week, was associated with a lower risk of caries in preschool children [17]. However, the evidence base for structured supervised-brushing programmes remains mixed. Santamaria et al., in a systematic review of school-based supervised-toothbrushing trials, found substantial heterogeneity in participant age, caries risk, and outcome measures across studies, precluding meta-analysis and preventing a definitive conclusion on the effectiveness of such programmes in reducing caries incidence in children and adolescents [18]. Our cross-sectional design cannot establish whether supervision itself is protective or simply a marker of broader parental engagement with oral health.

Dietary and access-related variables pointed in a similar direction. Sweets consumed before bedtime showed the strongest dietary association and remained a significant independent predictor, plausibly reflecting the reduced salivary clearance that occurs during sleep. Frequency of dental visits also remained an independent predictor, with less frequent attendance associated with higher odds of reported caries. This finding should nonetheless be interpreted with caution because attending the dentist mainly when problems arise may itself be shaped by a child already having experienced caries, so this predictor may partly capture health-seeking behaviour in response to prior symptoms rather than a purely preventive habit. A related concern is differential diagnostic opportunity: children who attend the dentist more often may simply have more chances to receive a caries diagnosis, independent of any underlying difference in disease burden. However, this mechanism would be expected to bias the association in the opposite direction to what was observed, since more frequent attendance would then predict more, not fewer, reported caries. The finding that less frequent attendance was associated with higher odds of reported caries argues against differential diagnostic opportunity as the primary explanation, though it cannot be fully excluded, particularly for children who rarely or never attend and for whom parents may be more likely to report caries status as unknown rather than absent. Consistent with this, the reason for the last dental visit followed the same pattern, with routine check-ups linked to markedly lower reported caries. This pattern may simply describe the same underlying tendency from two angles rather than indicating that visit type itself changes risk. A similar direction was reported by Camargo et al., who found that among preschool children, pain in the preceding six months predicted problem-driven visits, while routine check-ups were predicted instead by maternal education and prior preventive guidance, suggesting that the reason for attendance largely reflects pre-existing parental engagement rather than acting as an independent driver of oral health [19]. This is broadly consistent with Khairuddin et al., whose systematic review of longitudinal studies found that regular dental attendance was consistently associated with less caries experience and better oral health-related quality of life across five countries, although findings for decayed teeth specifically were less consistent across studies, a caveat that fits our own inability to disentangle attendance pattern from the broader preventive behaviours that likely accompany it [20].

A further pattern worth noting, though it should be interpreted cautiously given the descriptive nature of this section, is a possible gap between stated awareness and reported outcomes. Despite the dentist being the information source most associated with lower caries, most parents felt community prevention campaigns were insufficient, while participation in an educational program showed only a borderline association. This aligns with the general perception of dentists as the most trustworthy source of oral health information. Reyes Ortiz et al., in a cross-sectional survey of parents in Spain, found that dentists and paediatric dentists were rated among the most reliable sources consulted, ahead of most digital and social media channels [21]. This could suggest that general awareness-raising, without a clinical relationship behind it, may have limited practical effect, which is consistent with Peerbhay et al., whose scoping review of oral health promotion interventions in children and adolescents aged 8–18 found that traditional oral health education, while improving knowledge, is not an effective approach when used in isolation, without being paired with practical, reinforced, or clinically embedded components [22]. Our data cannot distinguish this from reverse causation, where parents of children with more dental problems may simply seek out more information after the fact.

Finally, the impact section produced the most consistent findings in the study, with all six assessed dimensions of parent-reported impact associated with caries and physical discomfort showing the largest effect size observed anywhere in the analysis. This mirrors findings from other paediatric and adolescent populations, where caries-specific impacts on eating, speaking, emotional state, and school performance have been shown to exceed those of other oral conditions such as gingival disease [23]. Similar patterns have been reported using parent-proxy instruments in preschool populations, where pain, difficulty eating, and reduced self-confidence were among the most frequently endorsed impacts of dental caries [24]. More broadly, the severity of caries experience has been shown to correlate with the extent of oral health–related quality-of-life impairment in children, reinforcing the interpretation that the strength of the impact findings in our sample reflects a genuine dose-related burden rather than a reporting artefact [25]. Taken together with the regression results, these findings tentatively support a picture in which caries risk in this population clusters around a small number of modifiable, everyday behaviours rather than being driven by any single dominant factor. This is consistent with the broader characterization of caries as a disease shaped by a multifactorial interplay of established and emerging determinants, in which dietary and behavioural influences remain central even as socioeconomic and biological factors also contribute [26]. Structural modelling approaches in other paediatric populations have similarly shown that oral hygiene practices act as a proximal pathway linking broader family and caregiver factors to children’s caries experience, supporting the interpretation that our independent predictors likely represent points of entry into a wider, interconnected pattern of risk rather than isolated causes [27].

Several limitations should be acknowledged. First, the cross-sectional design precludes causal inference. Associations such as those between dental visit type, awareness, and caries status may equally reflect reverse causation, whereby parents of children with more dental problems seek more care or information rather than these factors reducing caries risk. Second, and most importantly, the primary outcome of this study (reported caries status) was based entirely on parental report, with no clinical examination or linkage to dental records, even for a subsample. Because this outcome underlies both the reported prevalence and every association reported, from bivariate comparisons to the multivariable model, any misclassification would affect the entire analysis rather than a single variable in isolation. Hygiene behaviours and reported impact were subject to similar self-report limitations, introducing potential recall and social-desirability bias. The 11 “do not know” responses regarding caries diagnosis further indicate some uncertainty in parental knowledge of their child’s oral health. Third, participants were recruited through social media and parent-oriented groups, which may have favoured more digitally engaged, urban, and health-conscious respondents, limiting generalisability to the broader Romanian population; this is reflected in the unequal urban-rural split of the sample (78.5% vs. 21.5%). Fourth, the large number of bivariate tests performed, particularly for multiple-choice items analysed option-by-option, increases the risk of false-positive findings. No correction for multiple testing was applied. Bivariate results should therefore be interpreted as exploratory, with the multivariable model providing the more robust basis for identifying independent predictors. Fifth, age was entered into the multivariable model as an ordinal variable based on four clinically defined but unequally spaced categories reflecting stages of dental development, rather than as exact age. This assumes a comparable stepwise increase in risk between categories despite their differing width. The exact age was not collected and could therefore not be entered as a continuous variable, which would have been the preferred approach. Finally, the questionnaire did not collect data on parental education, family income, or other socioeconomic indicators repeatedly identified as caries risk factors in the literature, including in our own prior work in a related Romanian population [15]. This omission was intentional: the present instrument was designed to complement rather than duplicate that earlier socioeconomic-focused analysis, focusing instead on daily, modifiable behavioural and access-related exposures. Nonetheless, the absence of these indicators may partly explain the relatively modest proportion of variance explained by the regression model. Beyond model performance, some of the identified predictors, particularly rural residence and brushing supervision, may also partly reflect unmeasured socioeconomic differences between families rather than fully independent effects, and residual confounding by socioeconomic status cannot be ruled out.

## 5. Conclusions

In this sample of Romanian children and adolescents, reported dental caries was most consistently associated with the absence of parental supervision during brushing, an early and unsupported transition to independent brushing, sweets consumed before bedtime, infrequent or symptom-driven dental visits, and rural residence, factors that remained independent predictors even after mutual adjustment. The presence of reported caries was, in turn, associated with a substantial and consistent burden on the child’s daily functioning, spanning physical discomfort, functional limitation, embarrassment, social avoidance, and disrupted sleep and school performance. Taken together, these findings identify a small number of everyday, modifiable behaviours and access-related disparities most strongly associated with reported caries in this population. Whether targeting these specific factors, rather than generic awareness campaigns, would translate into reduced caries incidence remains to be tested in intervention studies.

## Figures and Tables

**Table 1:** Sociodemographic and clinical characteristics of the study sample.

Characteristic	Category	*n*	%
Age group	Under 6 years	185	36.4
	6–9 years	139	27.4
	10–12 years	73	14.4
	13–18 years	111	21.9
Sex	Male	276	54.3
	Female	232	45.7
Residence	Urban	399	78.5
	Rural	109	21.5
Reported caries	Yes	311	61.2
	No	186	36.6
	Do not know	11	2.2
Dental extractions	Yes	72	14.2
	No	434	85.4
	Do not know	2	0.4
Orthodontic appliance	Yes	55	10.8
	No	453	89.2

**Table 2:** Oral hygiene behaviours and their association with reported dental caries.

Variable	*n*	%	*p*	Cramér’s V
Toothbrushing frequency				
Twice a day or more	266	52.4	0.155 ns	0.116
Once a day	197	38.8		
A few times a week	38	7.5		
Rarely (a few times/month)	6	1.2		
Never	1	0.2		
Brushing duration				
Over 2 min	70	13.8	0.372 ns	0.093
1–2 min	229	45.1		
~30 s–1 min	164	32.3		
Under 30 s	33	6.5		
Do not know	12	2.4		
Brushing before bedtime				
Every evening, without exception	218	42.9	0.003 **	0.19
Almost every evening	189	37.2		
Occasionally (2–3×/week)	60	11.8		
Rarely (a few times/month)	23	4.5		
Very rarely (once/few months)	8	1.6		
Never	10	2.0		
Brushing supervision				
Parents	278	54.7	<0.001 ***	0.364
Older sibling/other family member	11	2.2		
Nobody, brushes alone	218	42.9		
Does not brush teeth	1	0.2		
Age started brushing alone				
Under 3 years	156	31.4	<0.001 ***	0.354
3–5 years	212	42.7		
6–8 years	65	13.1		
Over 8 years	15	3.0		
Still needs help	49	9.9		
Toothbrush type				
Manual	283	55.7	0.015 *	0.109
Electric	225	44.3		
Toothpaste type				
Fluoride	315	62.0	<0.001 ***	0.182
Non-fluoride	143	28.1		
Does not use toothpaste	5	1.0		
Do not know	45	8.9		
Toothbrush replacement frequency				
Every 3 months or more often	282	56.7	0.163 ns	0.115
Every 4–6 months	148	29.8		
Once a year	22	4.4		
When worn out	37	7.4		
Do not know/not changed regularly	8	1.6		
Rinsing with water/mouthwash after meals				
Yes, always	67	13.5	0.057 ns	0.123
Yes, sometimes	135	27.2		
Rarely (a few times/month)	77	15.5		
Never	218	43.9		
Perceived adequacy of oral hygiene habits				
Very adequate	209	42.1	0.004 **	0.164
Fairly adequate	239	48.1		
Inadequate	24	4.8		
Do not know	25	5.0		
Other oral hygiene methods used (multiple choice)				
Dental floss	111	22.3	0.312 ns	0.045
Mouthwash	142	28.6	<0.001 ***	0.167
Interdental brush	45	9.1	0.027 *	0.099
Does not use other methods	279	56.1	<0.001 ***	0.181

*p* and Cramér’s V from chi-square tests of each variable against reported caries diagnosis (Yes = 311, No = 186; “Do not know” excluded, *n* = 497). Multiple-choice item tested option-by-option (selected vs. not selected). *** *p* < 0.001; ** *p* < 0.01; * *p* < 0.05; ns = not significant.

**Table 3:** Dietary habits and their association with reported dental caries.

Variable	*n*	%	*p*	Cramér’s V
Sweets and unhealthy snacks				
Several times a day	94	18.5	<0.001 ***	0.231
Once a day	159	31.3		
A few times a week	173	34.1		
Rarely (a few times/month)	67	13.2		
Very rarely (once/few months)	10	2.0		
Never	5	1.0		
Sweetened beverages				
Daily	40	7.9	<0.001 ***	0.278
A few times a week	148	29.1		
Once a week	65	12.8		
Rarely (a few times/month)	145	28.5		
Very rarely (once/few months)	60	11.8		
Never	50	9.8		
Sweets before bedtime				
Regularly (every evening)	18	3.5	<0.001 ***	0.312
Occasionally (2–3×/week)	115	22.6		
Rarely (a few times/month)	118	23.2		
Very rarely (once/few months)	130	25.6		
Never	127	25.0		
Most frequent between-meal drink				
Water	448	88.2	0.009 **	0.175
Milk	11	2.2		
Natural sugar-free juice	15	3.0		
Carbonated soft drink	28	5.5		
Sweetened tea	4	0.8		
Unsweetened tea	2	0.4		
Fresh fruit and vegetables				
Several times a day	165	32.5	<0.001 ***	0.212
Once a day	163	32.1		
A few times a week	147	28.9		
Rarely (a few times/month)	27	5.3		
Very rarely (once/few months)	5	1.0		
Never	1	0.2		
Perceived diet influence on oral health				
Yes, very much	305	61.4	0.138 ns	0.105
Yes, to some extent	171	34.4		
Not at all	8	1.6		
Do not know	13	2.6		

*p*-values for frequency items (sweets and unhealthy snacks, sweetened beverages, sweets before bedtime, fresh fruit and vegetables) are from the Mann–Whitney U test; *p*-values for between-meal drink type and perceived diet influence are from chi-square tests. For all variables, Cramér’s V was computed from the Pearson chi-square statistic of the corresponding contingency table and is reported solely as a standardized effect-size measure, not as the basis for significance testing (*n* = 497). *** *p* < 0.001; ** *p* < 0.01; ns = not significant.

**Table 4:** Access to and use of dental services, and association with reported dental caries.

Variable	*n*	%	*p*	Cramér’s V
Routine check-up frequency				
Every 6 months	220	43.3	<0.001 ***	0.293
Once a year	138	27.2		
Less than once a year	34	6.7		
Only when having problems	77	15.2		
Does not go at all	39	7.7		
Timing of last visit				
Within last 6 months	335	65.9	<0.001 ***	0.307
Within last year	82	16.1		
Within last 2 years	37	7.3		
More than 2 years ago	11	2.2		
Never been	43	8.5		
Reason for last visit				
Routine check-up	304	59.8	<0.001 ***	0.426
Dental pain	42	8.3		
Caries treatment	90	17.7		
Other dental problems	72	14.2		
Dental fear				
Yes, always	61	12.0	0.017 *	0.128
Sometimes	125	24.6		
No, relaxed	322	63.4		
Perceived ease of access				
Very easy	314	61.8	0.515 ns	0.068
Fairly easy	148	29.1		
Difficult	39	7.7		
Very difficult	7	1.4		
Access to free public treatments				
Yes, frequently	86	16.9	<0.001 ***	0.264
Yes, occasionally	79	15.6		
No, but would need it	40	7.9		
No, never	303	59.6		
Reasons for not attending regularly (multiple choice)				
No perceived dental problems	244	49.1	<0.001 ***	0.264
High treatment costs	112	22.5	0.001 **	0.148
Child’s fear of the dentist	73	14.7	<0.001 ***	0.168
Lack of available time	66	13.3	0.067 ns	0.082
Difficult access to a dental office	17	3.4	0.853 ns	0.008
Other reasons	120	24.1	0.844 ns	0.009
Referral to specialist				
Yes	124	24.9	<0.001 ***	0.273
No	373	75.1		

*p* and Cramér’s V from chi-square tests against reported caries diagnosis (*n* = 497). Multiple-choice item tested option-by-option (selected vs. not selected). *** *p* < 0.001; ** *p* < 0.01; * *p* < 0.05; ns = not significant.

**Table 5:** Awareness and caries-prevention behaviours (Section V) and their association with reported dental caries.

Variable	*n*	%	*p*	Cramér’s V
Discussed oral health importance with child				
Yes, frequently	379	76.3	0.332 ns	0.083
Yes, occasionally	98	19.7		
Rarely	16	3.2		
Never	4	0.8		
Main source of oral health information (multiple choice)				
Parents/Family	459	92.4	0.012 *	0.113
Dentist	281	56.5	<0.001 **	0.211
School/Kindergarten	188	37.8	0.795 ns	0.012
Mass media (TV, internet, social media)	68	13.7	0.082 ns	0.078
Does not receive information on this topic	4	0.8	0.606 ns	0.023
Participation in educational program				
Yes	269	54.1	0.096 ns	0.113
No	129	26.0		
Do not know	61	12.3		
Never	38	7.6		
Perceived most effective education method (multiple choice)				
Interactive presentations at school/kindergarten	347	69.8	0.666 ns	0.019
Regular dentist visits	363	73.0	0.699 ns	0.017
Online educational campaigns (videos, apps)	132	26.6	0.120 ns	0.070
Printed materials (brochures, books)	115	23.1	0.833 ns	0.009
Family example and daily habits	361	72.6	0.152 ns	0.064
Sufficient prevention campaigns in community				
No	326	65.6	1.000 ns	0.001
Do not know	99	19.9		
Yes	72	14.5		
Interest in prevention program				
Yes	365	73.4	0.200 ns	0.080
Maybe, if easily accessible	98	19.7		
No	34	6.8		

*p* and Cramér’s V from chi-square tests against reported caries diagnosis (*n* = 497). Multiple-choice items tested option-by-option (selected vs. not selected). ** *p* < 0.001; * *p* < 0.05; ns = not significant.

**Table 6:** Parent-reported impact of dental problems on the child, and association with reported dental caries.

Variable	*n*	%	*p*	Cramér’s V
Functional limitations (chewing/speaking difficulty)				
No difficulty at all	402	80.9	<0.001 ***	0.304
Yes, difficulty chewing food	58	11.7		
Yes, difficulty speaking clearly	22	4.4		
Both of the above	15	3.0		
Physical discomfort (pain, last 3 months)				
No pain reported	366	73.6	<0.001 ***	0.349
Pain reported, and avoided certain foods	67	13.5		
Pain reported, but did not avoid foods	64	12.9		
Psychological disability (embarrassment)				
No such manifestations present	409	82.3	<0.001 ***	0.263
Felt embarrassed, but not less confident	46	9.3		
Felt embarrassed and less confident	42	8.5		
Social handicap (avoided smiling/speaking)				
No such problems occurred	442	88.9	<0.001 ***	0.208
Avoided smiling/speaking, without interaction difficulties	31	6.2		
Avoided smiling/speaking, with interaction difficulties	24	4.8		
Daily-activity impact: school performance				
No	442	88.9	0.001 **	0.177
Do not know	28	5.6		
Sometimes	15	3.0		
Yes	12	2.4		
Daily-activity impact: sleep problems				
No	395	79.5	<0.001 ***	0.194
Yes	45	9.1		
Sometimes	42	8.5		
Do not know	15	3.0		

*p* and Cramér’s V from chi-square tests against reported caries diagnosis (*n* = 497). *** *p* < 0.001; ** *p* < 0.01.

**Table 7:** Multivariable logistic regression for predictors of reported dental caries, with sensitivity analysis across alternative model specifications.

Predictor	Base Model—OR (95% CI)	“Do Not Know” → No Caries	“Do Not Know” → Caries	Parsimonious (5 Predictors)
Child age (per group)	1.84 (1.44–2.36) *	1.82 (1.43–2.32) *	1.82 (1.42–2.33) *	1.87 (1.46–2.39) *
Absence of brushing supervision	2.11 (1.22–3.63) *	2.14 (1.25–3.65) *	1.99 (1.16–3.40) *	2.02 (1.18–3.47) *
Sweets before bedtime	1.76 (1.44–2.15) *	1.73 (1.42–2.11) *	1.73 (1.42–2.11) *	1.78 (1.46–2.18) *
Frequency of dental visits	1.47 (1.23–1.76) *	1.66 (1.37–2.01) *	1.43 (1.19–1.72) *	1.61 (1.33–1.95) *
Rural residence	1.86 (1.06–3.25) *	1.63 (0.95–2.79)	1.96 (1.13–3.38) *	1.77 (1.02–3.09) *
Non-fluoride toothpaste	0.77 (0.48–1.22)	—	—	not included
Male sex	1.21 (0.78–1.87)	—	—	not included
*n*	497	508	508	497
Nagelkerke R^2^	0.361	0.369	0.345	0.365

Adjusted odds ratios with 95% confidence intervals. * *p* < 0.05. The base model (*n* = 497) reproduces the primary analysis (“Do not know” excluded); model χ^2^(7) = 152.69, *p* < 0.001. In the base model, child sex and non-fluoride toothpaste were retained but non-significant; only their base-model estimates are shown. OR = odds ratio; CI = confidence interval.

## Data Availability

The data presented in this study are available on request from the corresponding author. The data are not publicly available due to privacy and ethical restrictions, as the dataset contains information collected from parents regarding their children.

## References

[1] Do L.G., Song Y.H., Du M., Spencer A.J., Ha D.H. (2023). Socioecological Determinants of Child Oral Health—A Scoping Review. Community Dent. Oral Epidemiol..

[2] World Health Organization (2022). Global Oral Health Status Report: Towards Universal Health Coverage for Oral Health by 2030.

[3] World Health Organization (2019). Ending Childhood Dental Caries: WHO Implementation Manual.

[4] Watt R.G., Daly B., Allison P., Macpherson L.M.D., Venturelli R., Listl S., Weyant R.J., Mathur M.R., Guarnizo-Herreño C.C., Celeste R.K. (2019). Ending the Neglect of Global Oral Health: Time for Radical Action. Lancet.

[5] Large J.F., Madigan C., Pradeilles R., Markey O., Boxer B., Rousham E.K. (2024). Impact of Unhealthy Food and Beverage Consumption on Children’s Risk of Dental Caries: A Systematic Review. Nutr. Rev..

[6] Baghlaf K., Muirhead V., Moynihan P., Weston-Price S., Pine C. (2018). Free Sugars Consumption around Bedtime and Dental Caries in Children: A Systematic Review. JDR Clin. Trans. Res..

[7] Chou Y.C., Cheng F.S., Weng S.H., Tseng C.H., Hu H.Y., Liu C.H. (2025). Impact of Parental Health Beliefs on Early Childhood Caries: A Two-Year Longitudinal Study. Int. Dent. J..

[8] Xu X., Shi X., Xie X., Liu Y., Shi H., Wang Q., Wang J. (2025). Parental Health Belief and Behavioral Factors Influencing Oral Health Behaviors of Preschoolers in Shanghai: A Cross-Sectional Study. Sci. Rep..

[9] Alzahrani A.Y., El Meligy O., Bahdila D., Aljawi R., Bamashmous N.O., Almushayt A. (2024). The Influence of Parental Oral Health Literacy on Children’s Oral Health: A Scoping Review. J. Clin. Pediatr. Dent..

[10] Mousa M., Alkandari M., Alabbasi H., Alhujailan R., Bohamdi K., Ahmad L., Almatooq A., Alsulimmani W., Alhazmi H. (2026). Barriers to Dental Service Utilization in Children: A Systematic Review and Meta-Analysis. BMC Oral Health.

[11] Sabbagh H.J., Albeladi N.H., Altabsh N.Z., Bamashmous N.O. (2023). Risk Factors Associated with Children Receiving Treatment at Emergency Dental Clinics: A Case-Control Study. Int. J. Environ. Res. Public Health.

[12] Moca A.E., Țig I.A., Ciavoi G., Iurcov R., Șipoș L.R., Todor L. (2022). The Impact of the COVID-19 Pandemic on the Dental Emergency Service from Oradea, Romania: A Retrospective Study. Healthcare.

[13] Martins-Júnior P.A., Vieira-Andrade R.G., Corrêa-Faria P., Oliveira-Ferreira F., Marques L.S., Ramos-Jorge M.L. (2013). Impact of Early Childhood Caries on the Oral Health-Related Quality of Life of Preschool Children and Their Parents. Caries Res..

[14] Chimbinha Í.G.M., Ferreira B.N.C., Miranda G.P., Guedes R.S. (2023). Oral-Health-Related Quality of Life in Adolescents: Umbrella Review. BMC Public Health.

[15] Moca A.E., Țig I.A., Cherecheș J.O., Moca R.T., Iurcov R. (2025). Impact of Marital Status, Education, and Family Size on Parental Behaviors Toward Early Childhood Caries in Romania. Dent. J..

[16] Tavakol M., Dennick R. (2011). Making Sense of Cronbach’s Alpha. Int. J. Med. Educ..

[17] Castilho G.T., Pessoa M.N., de Oliveira C.C., de Melo L.S.A., Tagliaferro E.P.S., Pardi V. (2025). Family Factors Associated with Dental Caries Among 5-Year-Old Preschool Children. Front. Dent. Med..

[18] Santamaria R.M., Splieth C. (2018). Beneficial Effects of Supervised Toothbrushing on Caries Incidence in Children and Adolescents Are Questioned. Evid. Based Dent..

[19] Camargo M.B., Barros A.J.D., Frazão P., Matijasevich A., Santos I.S., Peres M.A., Peres K.G. (2012). Predictors of Dental Visits for Routine Check-Ups and for the Resolution of Problems Among Preschool Children. Rev. Saude Publica.

[20] Mohd Khairuddin A.N., Bogale B., Kang J., Gallagher J.E. (2024). Impact of Dental Visiting Patterns on Oral Health: A Systematic Review of Longitudinal Studies. BDJ Open.

[21] Reyes Ortiz A., Riolobos González M.F., García Navas Fernández de la Puebla L., Álvarez Alonso A., Cruz-Cruz F., Martín-Vacas A. (2026). Use of Digital Platforms and Social Media as a Source of Information on Children’s Oral Health by Parents: A Cross-Sectional Survey Analysis in a Spanish Sample. Front. Oral Health.

[22] Peerbhay F., Mash R., Khan S. (2025). Effectiveness of Oral Health Promotion in Children and Adolescents through Behaviour Change Interventions: A Scoping Review. PLoS ONE.

[23] Kaewkamnerdpong I., Urwannachotima N., Prasertsom P., Charoenruk N., Krisdapong S. (2023). Impact of Oral Diseases on 12- and 15-Year-Old Children’s Quality of Life: Condition-Specific Oral Health Related Quality of Life Analysis. BMC Oral Health.

[24] Pakkhesal M., Riyahi E., Naghavi Alhosseini A., Amdjadi P., Behnampour N. (2021). Impact of Dental Caries on Oral Health Related Quality of Life Among Preschool Children: Perceptions of Parents. BMC Oral Health.

[25] Alanzi A., Behzadi S., Alsumait A., Baskaradoss J. (2026). Is Oral Health-Related Quality of Life of Preschool Children Affected by the Severity of Early Childhood Caries?. Front. Oral Health.

[26] Issrani R., Alnusayri S., Alderaan D., Alruwaili S., Almufarrij R., Alkhershawy L. (2025). Risk Factors for Caries in Children and Adolescents: A Systematic Review. Open Dent. J..

[27] Qiu R.M., Lo E.C.M., Zhi Q.H., Zhou Y., Tao Y., Lin H.C. (2014). Factors Related to Children’s Caries: A Structural Equation Modeling Approach. BMC Public Health.

